# Soil fungal biodiversity and pathogen identification of rotten disease in *Aconitum carmichaelii* (Fuzi) roots

**DOI:** 10.1371/journal.pone.0205891

**Published:** 2018-10-31

**Authors:** Wei Wang, Dayan Zhang, Huan Wen, Qihao Wang, Cheng Peng, Jihai Gao

**Affiliations:** Pharmacy College, Chengdu University of Traditional Chinese Medicine, The Ministry of Education Key Laboratory of Standardization of Chinese Herbal Medicine, Key Laboratory of Systematic Research, Development and Utilization of Chinese Medicine Resources in Sichuan Province-Key Laboratory Breeding Base of Co-founded by Sichuan Province and MOST, Chengdu, China; Tallinn University of Technology, ESTONIA

## Abstract

*Aconitum carmichaelii*, commonly known as Fuzi, is a typical traditional Chinese medicine (TCM) herb that has been grown for more than one thousand years in China. Although root rot disease has been seriously threatening this crop in recent years, few studies have investigated root rot disease in Fuzi, and no pathogens have been identified. In this study, fungal libraries from rhizosphere soils were constructed by internal transcribed spacer (ITS) sequencing using the HiSeq 2500 high-throughput platform. A total of 948,843 tags were obtained from 17 soil samples, and these corresponded to 195,583,495 nt. At 97% identity, the libraries yielded 12,266 operational taxonomic units (OTUs), of which 97.5% could be annotated. In sick soils, *Athelia*, *Mucor* and *Mortierella* were the dominant fungi, comprising 10.3%, 10.1% and 7.7% of the fungal community, respectively. These fungi showed 2.6-, 1.53- to 6.31- and 1.38- to 2.65-fold higher enrichment in sick soils compared with healthy soils, and their high densities reduced the fungal richness in the areas surrounding the rotted Fuzi roots. An abundance analysis suggested that *A*. *rolfsii* and *Mucor racemosus*, as the dominant pathogens, might play important roles in the invading Fuzi tissue, and *Phoma adonidicola* could be another pathogenic fungus of root rot. In contrast, *Mortierella chlamydospora*, *Penicillium simplicissimum*, *Epicoccum nigrum*, *Cyberlindnera saturnus* and *Rhodotorula ingeniosa* might antagonize root rot pathogens in sick soils. In addition, *A*. *rolfsii* was further verified as a main pathogen of Fuzi root rot disease through hypha purification, morphological observation, molecular identification and an infection test. These results provide theoretical guidance for the prevention and treatment of Fuzi root rot disease.

## Introduction

*Aconitum carmichaelii* Debx. is a typical TCM herb that has been clinically used for almost two thousand years [1, 2]. Fuzi, the lateral roots of *A*. *carmichaelii* Debx., has been extensively used as a cardiotonic, analgesic, anti-inflammatory, and diuretic agent for the treatment of colds, polyarthralgia, diarrhoea, heart failure, beriberi, and oedema [3]. As a result, the government of China as well as various Chinese companies have highly invested in *A*. *carmichaelii* (Fuzi). More than two thousand hectares of Fuzi, which is considered an important daodi herb and economic crop, were cultivated in Jiangyou and Butuo in Sichuan Province in 2015. Some East Asian countries, such as Japan, Korea, Mongolia and India, import a large quantity of this traditional medicine from China.

However, large-scale planting encourages mortal threats, and the Fuzi crop yield per unit area has been reducing each year. For instance, ten years ago, 15,000 kg of Fuzi could be harvested per hectare, but only approximately 11,000 kg per hectare can be harvested at present. This decrease is due to root rot disease, which is difficult to remedy using traditional methods. Once one Fuzi plant is infected by the root rot pathogen, its leaves turn brown (or reddish purple) and droop, and its stems eventually wilt. In severe cases, strands of white hyphae are visible on the ground, and a reddish brown sclerotium forms approximately a week later. Underground lateral roots eventually show skin lesions and tissue decay. The normal infection speed of this disease can reach 0.5–1 metres per day. Plants in the same farm or farm located near the disease sources soon become sick, and this resulted in the infection of more than 50% of the area of Fuzi farmlands by root rot fungus in 2016.

Root rot is generally regarded as the primary disease caused by soil-borne pathogens, such as the genus *Fusarium* in cereal plants, *Rhizoctonia bataticola* in chickpea plants [4], *Phytophthora nicotianae* in pepper crops [5], and *Cylindrocarpon destructans* in *Panax ginseng* [6]. Other pathogens, such as *Magnaporthe grisea*, *M*. *oryzae*, *Colletotrichum graminicola*, *Leptosphaeria maculans* and *Cercospora beticola*, also infect the roots of their hosts to cause root rot [7]. Crop plants can display dry root rot, collar rot or black root rot disease under different environmental conditions [4], but the causes of root rot appear to vary. On the one hand, pathogens have been shown to exhibit great adaptability to various soils and hosts. For instance, the host range of *P*. *nicotianae* includes 255 plant genera in 90 families [5], and this pathogen can cause not only root but also collar rot in pepper plants [5, 8]. *C*. *destructans* can also cause disease symptoms in the roots of species other than *P*. *ginseng*, such as strawberry [6, 9] and grapevines [10]. On the other hand, root rot might derive from microbial imbalances in rhizosphere soils. Rotted chickpea roots exhibit a different microbial community structure compared with control roots [4], indicating that a balanced microbial community is extremely important in the maintenance of healthy plants.

Few studies have investigated the pathogens of Fuzi root rot, and neither the invasion process nor prevention and control methods are known. Moreover, the pathogens leading to Fuzi root rot have not yet been reported; therefore, the encouragement of fundamental research studies on Fuzi root rot is crucial for solving the problem of pathogen invasion. In this study, we used high-throughput sequencing technology to screen the fungal community in sick soils and identify potential pathogens for Fuzi root rot disease. In addition, the fungal colonies of sick roots were isolated, purified and identified based on ITS sequences. Additionally, a pathogen infection test was performed to determine which fungi could cause the disease. Through this research, we aim to provide theoretical guidance for the prevention and treatment of Fuzi root rot disease.

## Materials and methods

### Infected soils and Fuzi

In this study, sick soils refer to soils attached to Fuzi roots around which sclerotia emerge. Samples of rot-infected Fuzi and sick soils were harvested from Jiangyou in Sichuan Province, which is the core area of the *Aconitum* Good Agricultural Practice of Medicinal Plants and Animals (GAP) planting bases in China. In Jiangyou, the Da-Hua leaf-type Fuzi is the main type of Fuzi plants cultivated, and the Xiao-Hua leaf type was cultivated to a lesser degree. Compared with Xiao-Hua, Da-Hua leaf-type *Aconitum* has a higher yield, larger root tubers and weaker resistance to root rot disease.

The following four groups of root surface soils were obtained: healthy soils (T1-T5, combined to form G1), healthy Da-Hua Fuzi (D1-D4, combined to form G2), diseased Da-Hua Fuzi (DV1, DV2 and DV4, combined to form G3) and healthy Xiao-Hua Fuzi (X1-X5, combined to form G4). Each group of dry soil was approximately 300–500 g (100 g for each replicate), and 17 soil replicates were prepared respectively as described by Miao [11]. The samples of diseased Fuzi materials without soil were stored at 4°C prior pathogen isolation and purification. All backup samples used in the study were frozen at the State Bank of Chinese Drug Germplasm Resources, which is located at Chengdu University of Traditional Chinese Medicine.

### Soil genomic DNA extraction and PCR amplification

Genomic DNA was extracted from each soil using the CTAB reagent, and PCR was then performed using specific barcode primers. The reaction contained 15 μl of PCR Mix (2× Phusion® High-Fidelity PCR Master Mix with GC Buffer), 3 μl of each primer (2 μM), 2 μl of gDNA (1 ng/μl) and 10 μl of H_2_O. The reaction conditions were 98°C for 1 min, 30 cycles of 98°C for 10 s, 50°C for 30 s and 72°C for 30 s, and 72°C for 5 min, and the ITS primer sequences used were ITS5-1737F (5’-GGA AGT AAA AGT CGT AAC AAG G-3’) and ITS2-2043R (5’-GCT GCG TTC TTC ATC GAT GC-3’).

### cDNA library construction, species annotation and analysis

The TruSeq^®^ DNA PCR-Free Sample Preparation Kit was used to construct cDNA libraries of the fungal communities in the soil samples. After the libraries were quantified by qRT-PCR and Qubit 2.0, a HiSeq 2500 PE250 instrument was used to sequence their ITS nucleotides. The high-throughput data was analysed using Qiime (V1.7.0, http://qiime.org/scripts/). After removing primers, tags, adaptors and low-quality nucleotides, the MUSCLE programme (Version 3.8.31, http://www.drive5.com/muscle/) was used for sequence alignments to identify the phylogenetic relationships and representative sequences of all OTUs. The OTUs were assigned at the 97% identity level. The community composition of each soil sample was calculated at each classification level, including kingdom, phylum, class, order, family, genus and species. The OTU counts were normalized into relative abundances (%). Statistical analyses were conducted with SPSS (v19, http://www.spss.com.cn/), and curve fitting was performed with SigmaPlot (v. 12.5, www.sigmaplot.com/). Unless otherwise stated, *P* values ≤0.05 were considered significant.

### Diversity and community structure analysis

To evaluate the species richness of rhizosphere fungi in each soil sample, an α-diversity analysis was performed using Qiime software (Version 1.7.0) with the Shannon index.

The focus of this study was the differences in fungal diversity between sick and healthy soils. Qiime software was used for an unweighted pair-group method with arithmetic means (UPGMA) analysis based on the UniFrac distance. The soil groups were clustered in a weighted UniFrac tree based on their relative fungal abundances at the phylum level. A principal component analysis (PCA) of the soil fungi in the four groups of soils was performed using the ade4 function in R software (Version 2.15.3).

### Isolation and identification of rot-disease pathogens in Fuzi

Fresh samples of rotted Fuzi were washed and sterilized with 75% ethanol. The interior milky tissue was sliced into 0.5-cm^2^ pieces, and the square blocks of Fuzi were incubated on PDA medium (200 g of potato, 30 g of glucose, 5 g of agar, 1 g of MgSO_4_, and 5 g of KH_2_PO_4_) at 32°C. Once the hyphae grew to approximately 2–3 cm, they were purified three times.

ITS barcode PCR, sequencing and nucleotide blast analysis () were performed to identify the pathogens isolated from rotted Fuzi tissues. The DNA of the pathogens was extracted using a Plant Fungal DNA Extraction Kit with the ITS sequence primers ITSlF (5’-CTT GGT CAT TTA GAG GAA GTA A-3’) and ITS4B (5’-CAG GAG ACT TGT ACA CGG TCC AG-3’). The PCR reactions contained 25 μl of PCR Mix (SsoFast TM EvaGreen® Supermix), 2 μl of ITS1F, 2 μl of ITS4B, 1 μl of gDNA and 20 μl of ddH_2_O. The reaction programme was 94°C for 3 min, 35 cycles of 94°C for 40 s, 55°C for 40 s, and 72°C for 90 s, and 72°C for 5 min. Sequencing was finished by the Chengdu branch of Biological Science and Technology Co., Ltd.

After ITS sequence alignment with the NCBI database, the pathogens in rotted Fuzi tissue were identified with homology >90%. A phylogenetic tree was then constructed based on the neighbour-joining (NJ) method using MEGA 4.0 software (http://www.megasoftware.net/) with the default parameters.

### Pathogenic infection

To detect the invasion effects of candidate rot pathogens, 0.5 cm^2^ of healthy fresh Fuzi was inoculated onto candidate mycelia of purified pathogens and cultured in the dark at 32°C. The infection status of the Fuzi materials and their morphology and colours were observed and compared with those of diseased aconite in the field.

## Results and discussion

### Fuzi root rot disease

**Fig 1:**
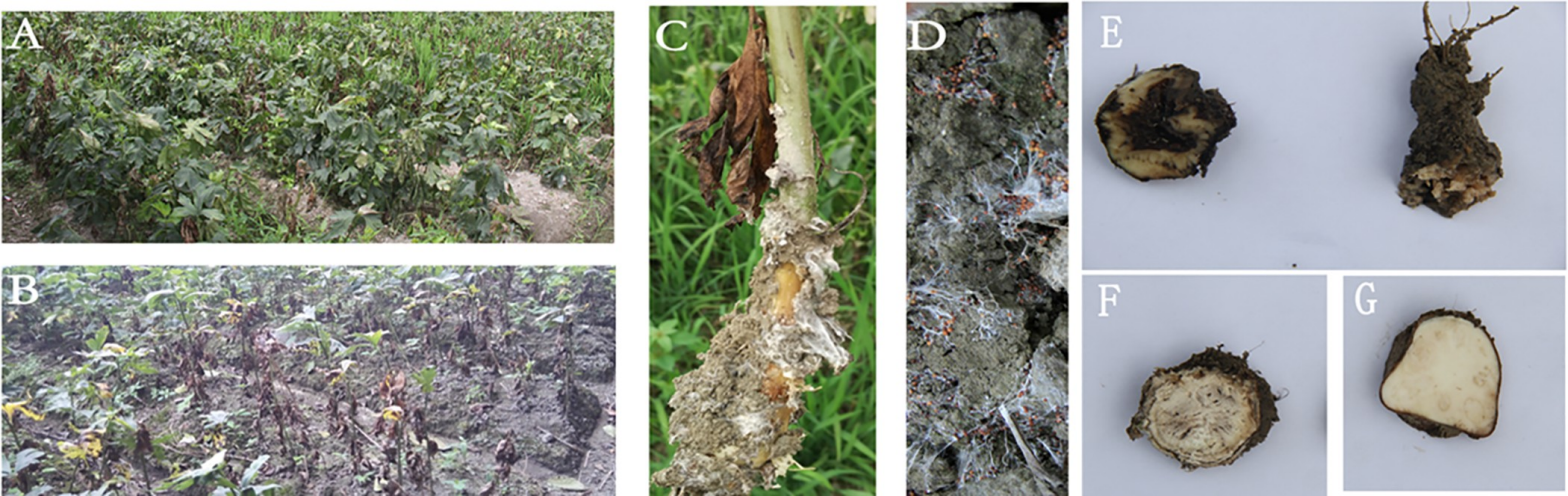
Diseased *Aconitum carmichaelii* (Fuzi) plants.

Diseased *Aconitum carmichaelii* plants were observed in this study (Fig 1). Once the plants were infected with root rot disease, which is also called “southern blight”, the lateral root tissues of Fuzi plants became soft from the outside to the inside, with the root skins rotting first and the hairy roots falling off. At the later stages of the disease, the roots emitted a foul smell, and white hyphae were observed on their surfaces. Moreover, the upper parts of *A*. *carmichaelii* plants wilted, and the leaves turned brown (or reddish purple) and dropped from the plant starting from the bottom. In addition, white hyphae were distributed unevenly in the soil, usually in visible clusters, and reddish-brown sclerotia arose and gathered near the soil surface (this type of soil is denoted “sick soil” in the manuscript). The series of symptoms observed was similar to that described in previous reports [12, 13].

### Construction of the soil fungal database

To explore the pathogenesis of Fuzi root rot disease, 17 soil replicates from four group of samples were used to construct fungal databases. In total, high-throughput sequencing produced 996,653 reads from the 17 soil samples. After removing the barcode primers, these were combined into 975,213 reads, and 948,843 ITS tags (95.25%) were obtained after the QC and Nochime analyses, yielding a total of 195,583,495 nt and an average length of 206 nt for each tag (S1 Table.). The Q30 value was 99.36% after filtrating the chimeric sequences in effective tags, indicating that the fungal databases were of high quality. The high-throughput sequencing data were uploaded to the NCBI database under Accession Number SRP142412.

**Fig 2:**
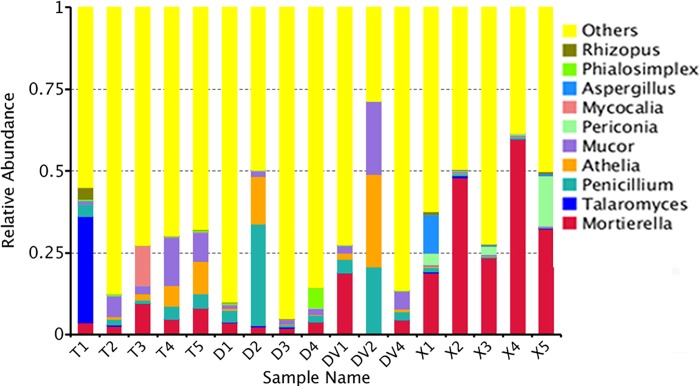
Relative abundance of rhizosphere fungi around Fuzi roots at the genus level.

At 97% identity, the fungal libraries yielded 12,266 clusters, also known as OTUs, of which 9198 (75%) were unique, and each soil sample provided an average of 722 OTUs (S1 Fig). For annotation analysis, 39.5–97.5% of fungi were classified into seven taxonomic levels (species, genus, family, order, class, phylum and kingdom) (S2 Fig). The soil fungi were mainly distributed in the Zygomycota (38.3%), Ascomycota (31.3%), Basidiomycota (28.2%) and Chytridiomycota (0.5%) phyla, and the genus-level analysis classified most of the soil fungi as *Mortierella*, *Talaromyces*, *Penicillium*, *Athelia* and *Mucor* (Fig 2 and Table 1).

**Table 1:** Relative abundance of rhizosphere fungi at the genus level.

Genus	Relative abundance in rhizosphere soils (%)			
	G1	G2	G3	G4
*Mortierella*	0.05627±0.0294	0.029317±0.0089	0.077484±0.0977	0.364652±0.0172
*Talaromyces*	0.066607±0.0015	0.00192±0.0013	0.000812±0.0005	0.002565±0.0011
*Penicillium*	0.029211±0.0085	0.094064±0.0145	0.09101±0.0784	0.006624±0.0045
*Athelia*	0.038747±0.0084	0.038703±0.0072	0.10287±0.0856	0.001846±0.0008
*Mucor*	0.066228±0.0554	0.015694±0.0044	0.100743±0.1006	0.003539±0.0005
*Periconia*	0.000752±0.0003	0.000996±0.0001	0.000048±0.0000	0.044616±0.0231
*Mycocalia*	0.025143±0.0238	0.000268±0.0002	0.000014±0.0000	0.000244±0.0002
*Aspergillus*	0.00038±0.0002	0.000243±0.0002	0.000124±0.0001	0.026304±0.0112
*Phialosimplex*	0.002945±0.0018	0.016984±0.0089	0.001149±0.0010	0.000219±0.0002
*Rhizopus*	0.007578±0.0016	0.000294±0.0001	0.000076±0.0000	0.003705±0.0033
Others	0.706139±0.1167	0.801516±0.2072	0.62567±0.3017	0.545687±0.1307

### Fungal diversity of four soil groups

**Fig 3:**
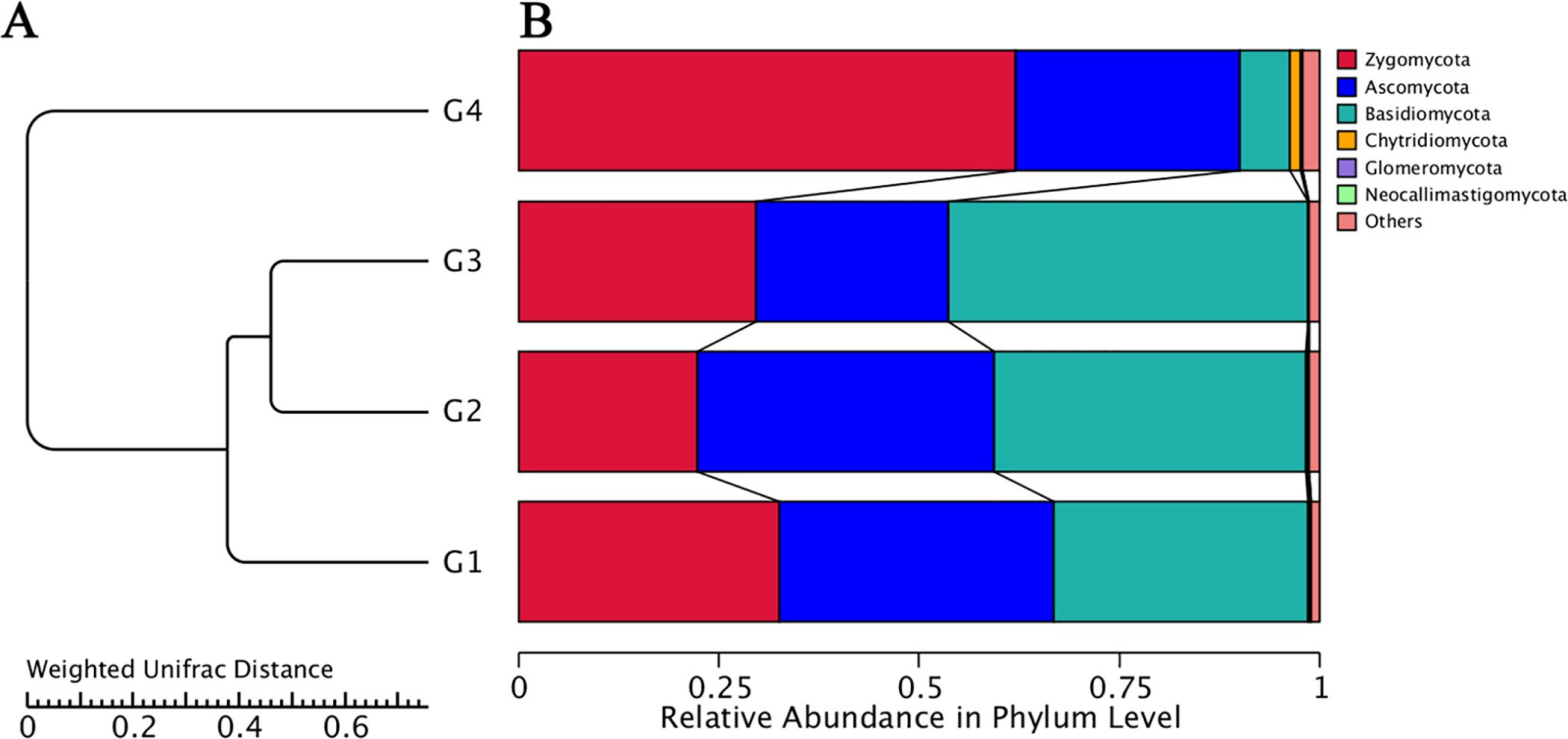
UPGMA cluster tree (A) and relative abundance (B) of rhizosphere fungi around Fuzi roots at the phylum level.

For the diversity analysis, a visualized UPGMA tree was constructed with the fungi in sick (G3) and healthy soils (G1, G2 and G4). The results revealed various diversities at every taxonomic level. At the phylum level, G3 contained the least abundances of Chytridiomycota, Ascomycota and Zygomycota fungi but the highest abundance of Basidiomycota fungi. The fungi in the G4 samples, which were obtained from rot-resistant Fuzi, mostly included Chytridiomycota, Zygomycota and Glomeromycota, with a lower abundance of Basidiomycota (Fig 3).

**Fig 4:**
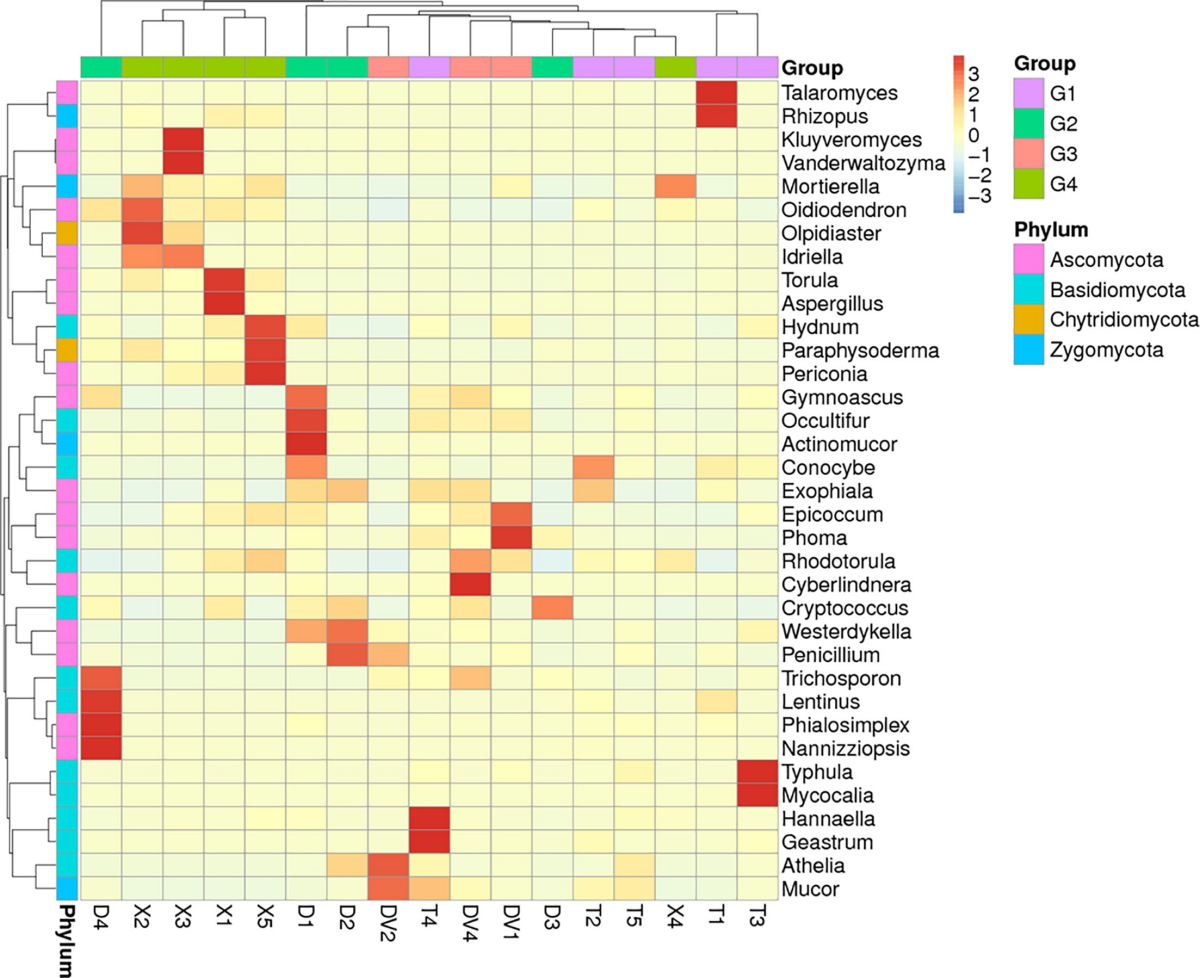
Heat map of the abundance of rhizosphere fungi around Fuzi roots at the genus level.

**Fig 5:**
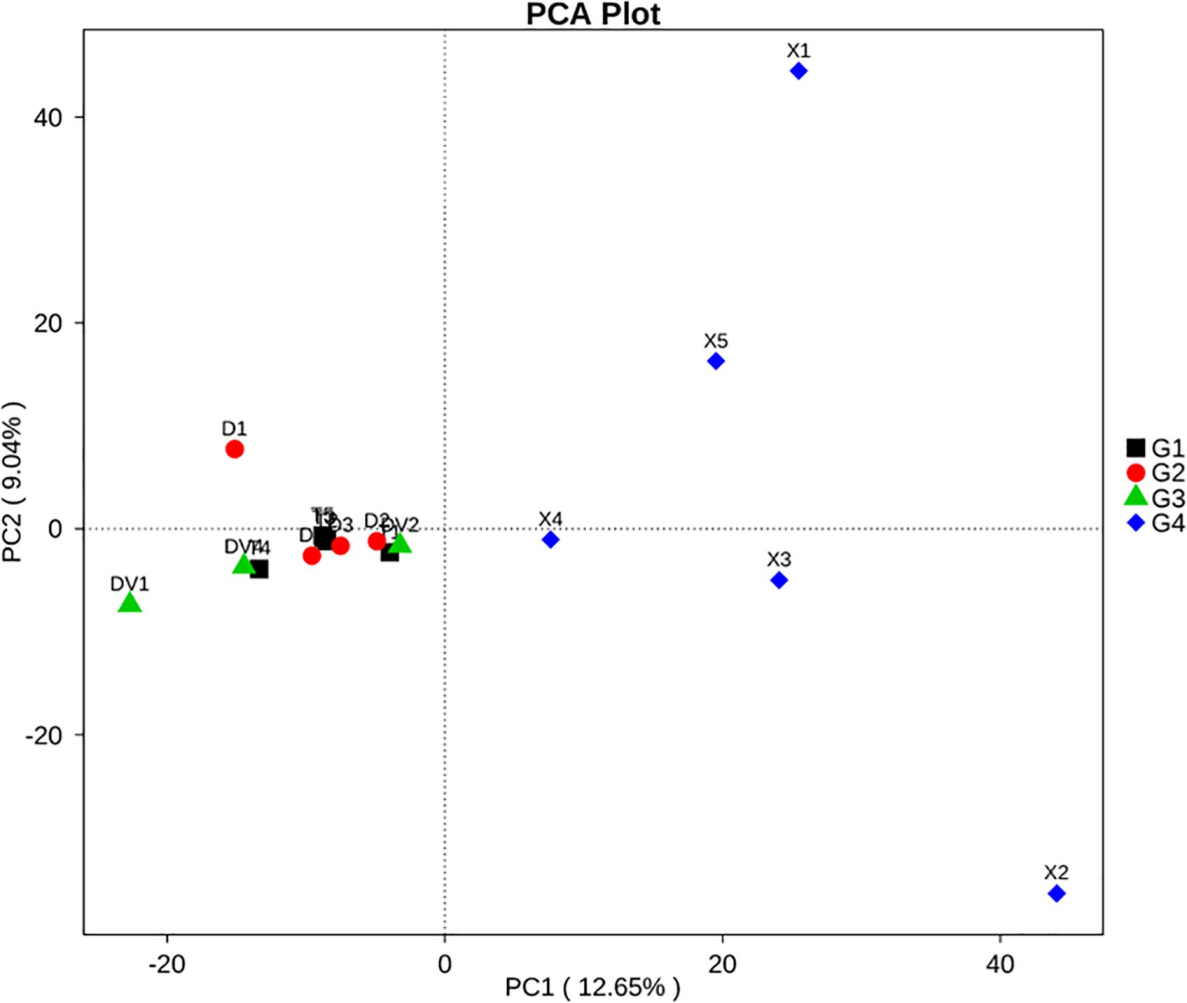
Variation in the fungal composition of the four soil groups determined by PCA.

At the genus level, the healthy soils (G1, healthy) showed the highest species richness (Shannon value = 5.32), and the fungal community was mainly composed of *Talaromyces* (6.66%), *Mucor* (6.62%), *Mortierella* (5.63%), and *Athelia* (3.87%) (Fig 4 and Table 2). The rhizosphere soils (G2 and G3) collected from areas surrounding Da-Hua Fuzi plants shared similar richness values (4.91 and 4.76, respectively) and some common dominant fungi, such as *Penicillium* (9.4% in G2 and 9.1% in G3). However, the *Athelia* content in sick soils (G3) contributed 10.3% of the fungal biomass, which is 2.6-fold higher than that found for G2. In addition, the abundances of *Mucor* and *Mortierella* in sick soils were 1.6- and 5.41-fold higher than those in G2, respectively (Fig 4 and Table 2). High densities of dominant fungi reduced the biodiversity of G3 in comparison to G2 and G1. *Mortierella* made up 36.4% of the fungal community in Xiao-Hua Fuzi plants (Fig 4 and Table 2), which decreased the richness value (Shannon value = 4.46) of G4 soils. The results were supported by the PCA results of the four groups of soil fungi (Fig 5). In particular, the PCA results showed that the fungal structure of G4 soils was far from those of G1-G3, echoing the results of the phylum-level diversity analysis of rhizosphere fungi around Fuzi roots (Fig 3). Based on the rot-resistant feature of Xiao-Hua Fuzi, an in-depth study of the fungal community of G4 soils might shed light on the biological prevention and control for Fuzi root-rot disease.

**Table 2:** Relative abundance of rhizosphere fungi around Fuzi roots.

Fungus species	Relative abundance in rhizosphere soils (%)			
	G1	G2	G3	G4
*Mortierella alpina*	0.003721±0.0008	0.005126±0.0049	0.001115±0.0005	0.289409±0.1870
*Talaromyces purpureogenus*	0.065368±0.0443	0.001136±0.0010	0.000138±0.0001	0.001379±0.0013
*Penicillium simplicissimum*	0.024462±0.0145	0.086455±0.0778	0.073272±0.0680	0.004989±0.0038
*Athelia rolfsii*	0.038677±0.0221	0.038367±0.0209	0.102828±0.0956	0.001797±0.0008
*Mucor racemosus*	0.059967±0.0479	0.014398±0.0045	0.093584±0.0734	0.001925±0.0007
*Mortierella chlamydospora*	0.041382±0.0312	0.010645±0.0058	0.07352±0.0580	0.001879±0.0006
*Periconia byssoides*	0.000752±0.0003	0.000996±0.0006	0.000048±0.0000	0.044616±0.0331
*Mycocalia denudata*	0.025143±0.0138	0.000268±0.0002	0.000014±0.0000	0.000244±0.0002
*Aspergillus tamarii*	0.000211±0.0001	0.000134±0.0001	0.000007±0.0000	0.02601±0.0110
*Phialosimplex caninus*	0.002912±0.0018	0.016984±0.0099	0.001149±0.0010	0.000219±0.0002
*Mortierella ambigua*	0.000756±0.0002	0.001482±0.0004	0.000213±0.0002	0.03273±0.0244
*Mortierella oligospora*	0.001161±0.0004	0.000661±0.0008	0	0.026704±0.0222
*Rhizopus arrhizus*	0.007529±0.0016	0.000263±0.0001	0.000076±0.0000	0.002817±0.0027
*Conocybe macrospora*	0.002577±0.0011	0.008151±0.0045	0.000255±0.0001	0.000496±0.0002
*Olpidiaster brassicae*	0.000322±0.0001	0.000196±0.0001	0.000096±0.0000	0.009115±0.0073
*Conocybe coprophila*	0.008541±0.0053	0.000780±0.0005	0.000028±0.0000	0.000058±0.0000
*Hannaella oryzae*	0.006088±0.0041	0.000909±0.0006	0.000847±0.0006	0.001747±0.0011
*Mortierella hypsicladia*	0.005555±0.0020	0.008900±0.0017	0.002154±0.0011	0.000525±0.0004
*Mortierella capitata*	0.000376±0.0003	0.000160±0.0001	0	0.007603±0.0034
*Kluyveromyces hubeiensis*	0.000045±0.0000	0.00001±0.0000	0.000014±0.0000	0.004328±0.0016
*Penicillium oxalicum*	0.000946±0.0005	0.001223±0.0010	0.006925±0.0018	0.001218±0.0010
*Lentinus squarrosulus*	0.001627±0.0009	0.005152±0.0011	0.000021±0.0000	0.000021±0.0000
*Penicillium solitum*	0.000206±0.0002	0.000284±0.0002	0.006814±0.0011	0.000025±0.0000
*Mortierella exigua*	0.000644±0.0002	0.000191±0.0000	0.000034±0.0000	0.005513±0.0039

### Candidate pathogens of fuzi root rot disease

To identify pathogens of Fuzi root rot disease, the fungal species enriched in sick soils were targeted. In the rotted Fuzi rhizosphere soils (G3), *A*. *rolfsii*, *Mucor racemosus*, *Mortierella chlamydospora* and *P*. *simplicissimum* were detected at levels higher than 5% (Table 2 and Fig 4). *A*. *rolfsii*, also called *Sclerotium rolfsii*, is a facultative plant pathogen and the causal agent of “southern blight” disease in crops such as potato, soybean, sunflower and some ornamental plants [14, 15, 16]. This pathogen is confined to warm and moist territories (such as Jiangyou in China) with daily average air temperatures of 30–33°C in mid-summer. In this study, the obvious rot morphology of DV2 Fuzi (Fig 1) and its dominant fungus in rhizosphere soil (Fig 4) demonstrated that *A*. *rolfsii* is one of the pathogens of Fuzi root rot disease. *Mucor racemosus* has been reported to be a causative pathogen of soft rot in cherry tomato [17]. *Mortierella* is ubiquitous in rhizosphere soils from a range of temperate and tropical forests and agricultural habitats [11]. This fungus usually plays a role in the maintenance of the micro-ecological balance by suppressing soil-borne pathogens through competition for nutrients and by assisting host plants with phosphorus/nitrogen absorption. *P*. *simplicissimum* is a plant growth-promoting fungus that induces plant resistance through the activation of multiple defence signals [18].

Some fungi that were not identified as the main constituent species in sick soils (G3) were nevertheless found to be enriched in these soils compared with healthy soils. For example, *Phoma adonidicola* and *Epicoccum nigrum* in DV1 and *Cyberlindnera saturnus* and *Rhodotorula ingeniosa* in DV4 are shown in Fig 4. Many fungi belonging to the *Phoma* genus are the causal agents of brown root rot, which causes severe root lesions in alfalfa, soybean and other perennial forage legumes [14]. This phenomenon might also be true for Fuzi plants, whose rotted-root annexed soil (DV1) had 20- to 40-fold higher amounts of *P*. *adonidicola* than healthy soils (T1-T5). It is noteworthy that the other three fungi that were enriched in sick soils appear to play competitive and antagonistic roles against the rot pathogens in Fuzi. A few *E*. *nigrum* isolates have been reported to significantly reduce the radial growth of both *Pythium debaryanum* and *P*. *ultimum* [19]. A *Cyberlindnera* fungus has been proven to exhibit antagonistic activities against a range of pathogenic and saprophytic filamentous fungi [20]. Additionally, *Rhodotorula* can protect sugar beets against the soil pathogen *Rhizoctonia solani* [21, 22]. Thus, these fungi might have potential as a novel bio-control agent against Fuzi pathogenic fungi.

These findings suggest that *A*. *rolfsii* and *Mucor racemosus*, as the dominant pathogens, play important roles in the invasion of Fuzi tissue and the induction of root rot symptoms, and increases in the *P*. *adonidicola* abundance would worsen the disease. In contrast, *M*. *chlamydospora*, *P*. *simplicissimum*, *E*. *nigrum*, *C*. *saturnus* and *R*. *ingeniosa* might antagonize the pathogens in sick soils around Fuzi roots.

### Separation and identification of the root rot pathogen

**Fig 6:**
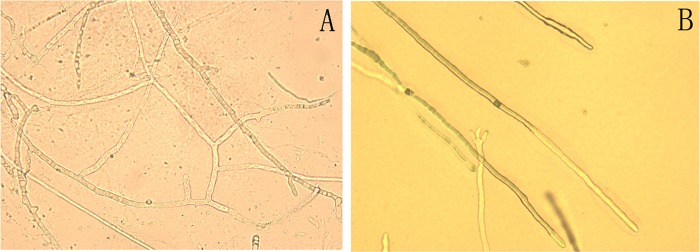
Hyphal morphology of suspected pathogens causing Fuzi root rot disease.

**Fig 7:**
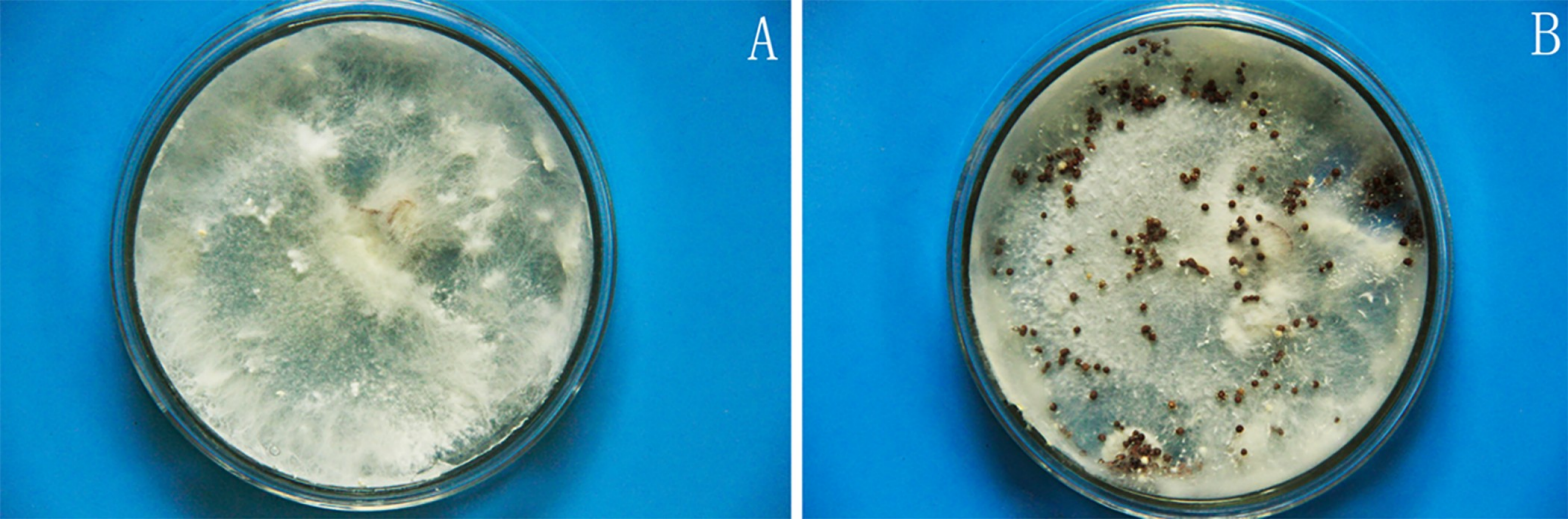
Cultivation of suspected pathogenic fungus (A) and its sclerotium (B) in PDA medium.

In this study, the rotten tissue of diseased Da-Hua Fuzi plants was used for pathogen isolation, and one strain of white fungus was obtained after purification. Its white, slender and crossed hyphae spread rapidly in the PDA medium, as a rate of approximately 2–3 cm per day (Fig 6). The fungal morphology observed under a microscope closely matched that observed in other studies [23]. After one week, brown and black sclerotia appeared in the culture dishes, and the hyphae showed slight shrinkage (Fig 7).

**Fig 8:**
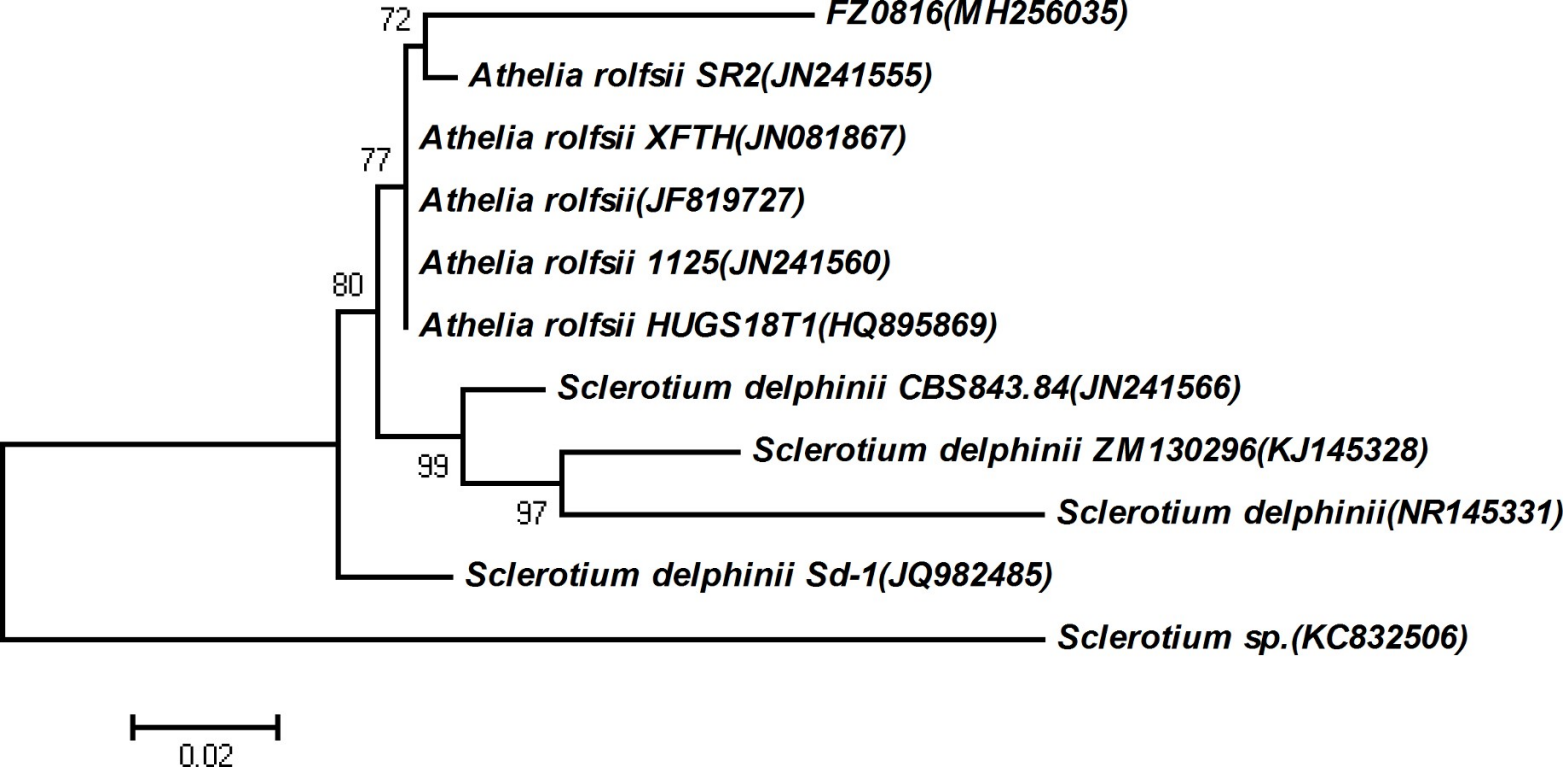
Phylogenetic tree of *A*. *rolfsii* strains.

In addition to morphological verification, the ITS barcode method was used for the identification of fungal species. The ITS fragment of fungus isolated from rotten Fuzi plants was found to be highly similar to sequences of known *A*. *rolfsii* strains in the NCBI database (S3 Fig). Then, the construction of a phylogenetic tree revealed that the fungus was closely related with other strains of *A*. *rolfsii* (Fig 8), indicating that it differs from known strains. These results verified previous inferences that *A*. *rolfsii* is the main pathogen causing Fuzi root rot disease. The newly discovered *A*. *rolfsii* was named FZ0816 and submitted to the NCBI database under Accession Number MH256035.

### Fuzi Infection by the suspected pathogen

To detect the invasion effects of the suspected pathogenic fungi FZ0816, fresh blocks of healthy Fuzi tissue were inoculated onto candidate mycelia at 32°C in the dark. After three days of fungal growth, its white hyphae became attached to the Fuzi tissue surface, and the tissue blocks began to rot and give off a bad smell. All the symptoms of the tested Fuzi tissues were extremely similar to those of diseased Da-Hua Fuzi plants in the wild. These results verify previous inferences that *A*. *rolfsii* is the main causative pathogen of Fuzi root rot disease.

## Supporting information

S1 Fig OTU cluster analysis of rhizosphere fungi in 17 soil samples. (TIF)

S2 Fig Fungal distribution of the annotation results obtained at seven taxonomic levels. (TIF)

S3 Fig Sequence alignment of *A*. *rolfsii* strains. (TIF)

S1 Table Quality of the high-throughput sequencing of fungi in rhizosphere soils around Fuzi roots. (XLSX)

## Abbreviations

TCM: typical traditional Chinese medicine
ITS: internal transcribed spacer
OTUs: operational taxonomic units
GAP: Good Agricultural Practice of Medicinal Plants and Animals
UPGMA: Unweighted Pair-group Method with Arithmetic Means
NJ: Neighbour-Joining
