# Supplementary figures and images for: Soil fungal biodiversity and pathogen identification of rotten disease in Aconitum carmichaelii (Fuzi) roots
Source: PLoS One. 2018 Oct 31;13(10):e0205891. doi: 10.1371/journal.pone.0205891 (PMC6209216; doi:10.1371/journal.pone.0205891)

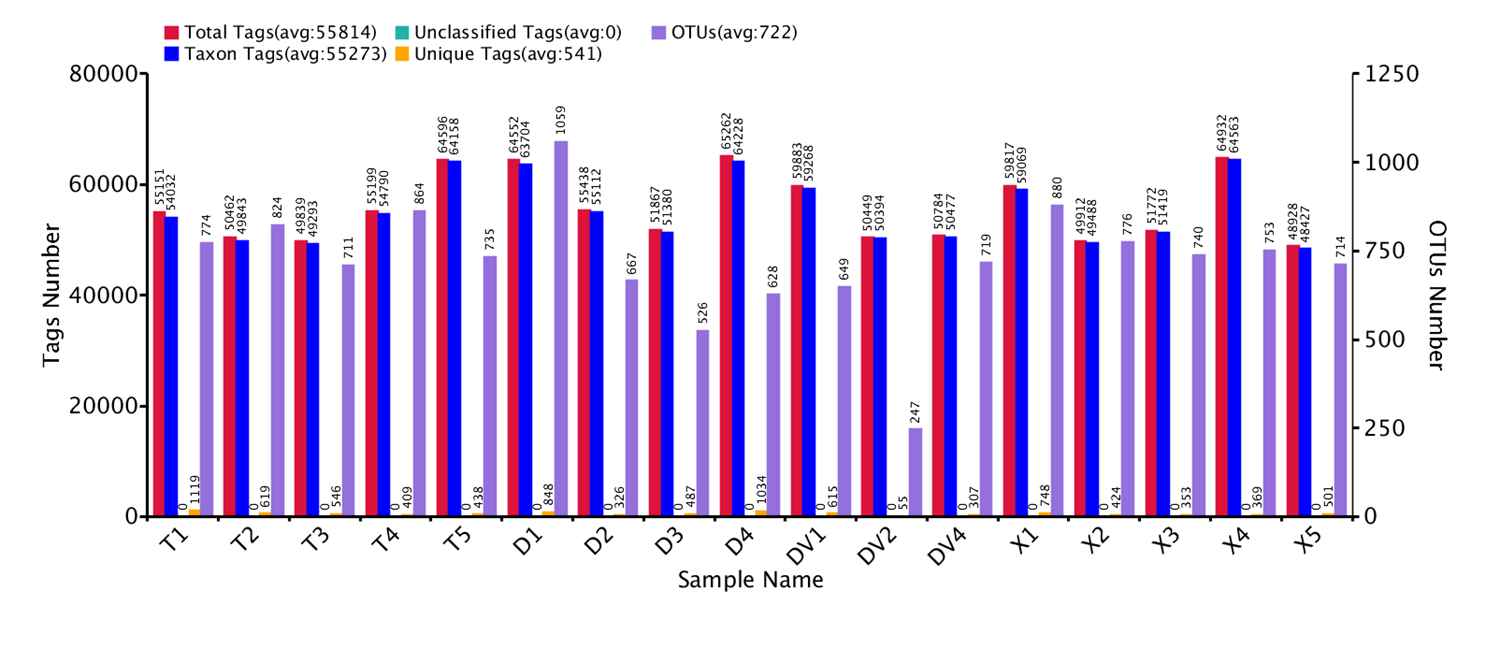

Supplement: S1 Fig — (TIF) [file pone.0205891.s001.tif]

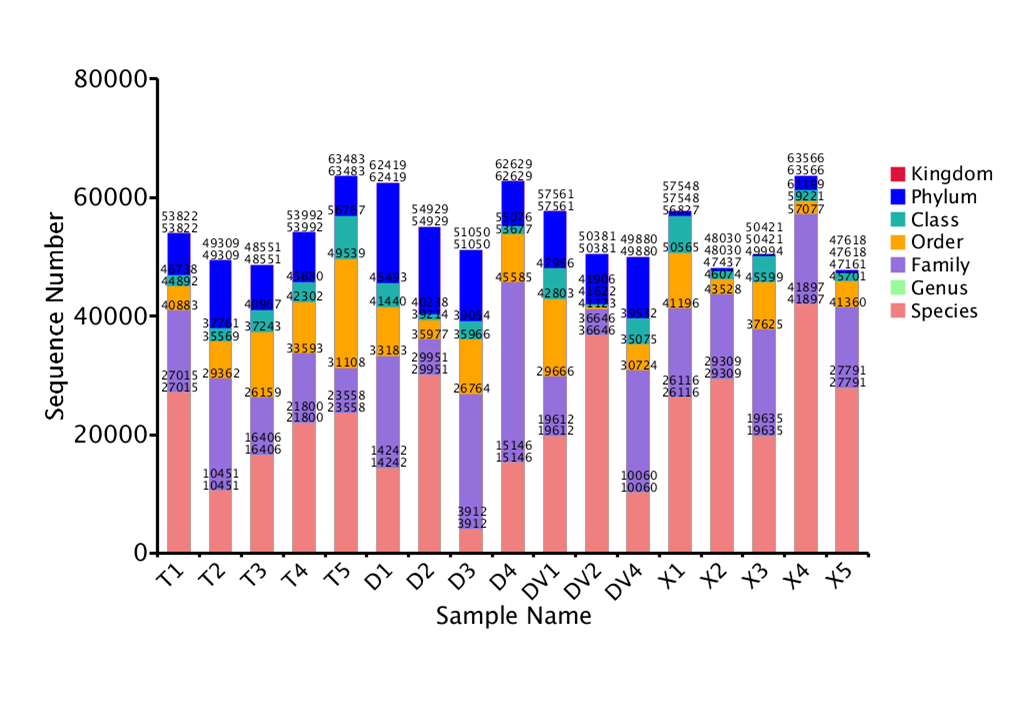

Supplement: S2 Fig — (TIF) [file pone.0205891.s002.tif]

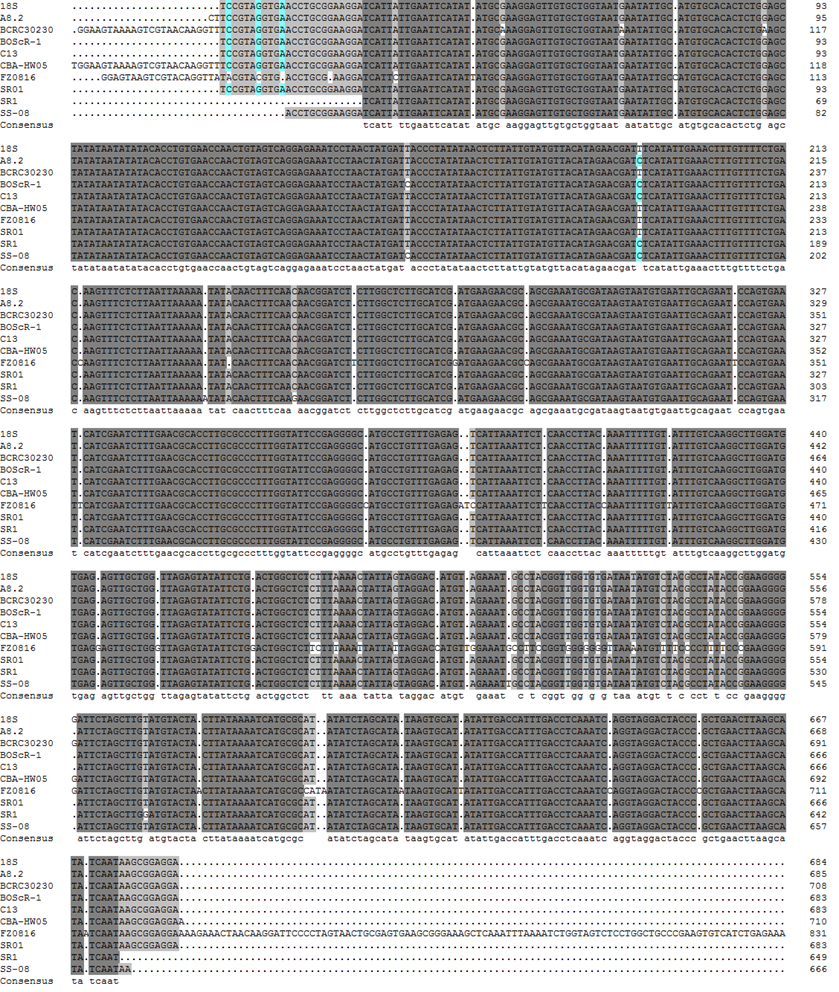

Supplement: S3 Fig — (TIF) [file pone.0205891.s003.tif]
